# Letter to editor on “Deep learning facilitated discovery of prognosis biomarkers and their ligands to improve liver cancer treatment”

**DOI:** 10.1097/JS9.0000000000003656

**Published:** 2025-11-05

**Authors:** Jia Tang, Mi Yan, Zhangxue Hu

**Affiliations:** Department of Pediatrics, Army Medical Center, Daping Hospital, Army Medical University, Chongqing, China

*Dear Editor,*

We have read with great interest the article by Wang *et al* titled “Deep learning facilitated discovery of prognosis biomarkers and their ligands to improve liver cancer treatment” published in your journal^[1]^. The author proposes a deep learning-based framework (DLCP) that integrates multiple omics data to stratify hepatocellular carcinoma (HCC) patients, identify prognostic biomarkers, and recommend potential therapeutic ligands. This study emphasizes the potential of artificial intelligence in advancing precision oncology, particularly by identifying RAC1 as a key biomarker and KGA-1083b as a promising ligand. Although this work represents an important step forward, several limitations should be considered to provide background for its contribution and guide future research. Due to the uniqueness of the team, its universality is limited.

## The model was mainly trained and validated on TCGA-LIHC and LIRI-JP queues

In terms of data, they used TCGA data, but TCGA liver cancer data mainly come from Western populations, which may not be suitable for Asian populations? In Asia, liver cancer is mainly associated with hepatitis B, while in the West, liver cancer is mainly associated with alcoholic and nonalcoholic fatty liver disease. The LIRI-JP queue they validated comes from the Japanese population and is also related to the virus, which may limit the generalizability of the results. Future research should include cohorts of different etiologies to ensure broader applicability.

## Lack of integration between proteomics and spatial genomics

Although DLCP integrates genomic, transcriptomic, and epigenetic data, it does not include proteomics or phosphoproteomics data, which are crucial for understanding the dynamics of functional protein levels. In addition, spatial transcriptomics or various immunohistochemical data can provide key insights into the heterogeneity of the tumor microenvironment, which cannot be captured by block omics data.

## Interpretability and biological rationality of DL features

Although deep neural networks have identified marker genes and methylation sites related to survival, the biological mechanisms behind these features remain largely unexplored. If there is no functional validation (such as CRISPR screening or pathway analysis), the causal role of these features in HCC progression remains speculative.

## Scope of experimental verification

Although the author validated the expression of RAC1 in mice and performed SPR/CETSA ligand binding, the in vivo efficacy of KGA-1083b was not evaluated. Animal models and metastasis models of HCC with genetic diversity will provide stronger evidence for their therapeutic potential^[2]^.

## Comparison with existing multi-component integration methods

This study did not comprehensively compare DLCP with other state-of-the-art multiomics ensemble methods, such as MOFA+, DeepSurv, PathCNN, and other deep learning models. This comparison will strengthen the claim of outstanding performance^[3]^.

## Clinical translation and drug development challenges

Although KGA-1083b has shown promising binding affinity and anti-HCC activity in vitro, its pharmacokinetics, toxicity, and bioavailability are still uncertain. Due to poor solubility, stability, and synthetic scalability, natural product-derived compounds often face challenges in drug development.

## Conclusion

Wang *et al* provided a valuable computational framework that emphasizes the power of deep learning in oncology. However, the above limitations indicate that it is necessary to further validate DLCP in different cohorts, integrate additional omics layers, and conduct thorough functional and pharmacological testing before considering clinical implementation. We encourage authors to address these issues in future research to enhance the impact and practicality of their promising frameworks.

Artificial INtelligence – the TITAN guideline^[4]^.

## Data Availability

Not applicable.
